# Concrete Strength Prediction Using Different Machine Learning Processes: Effect of Slag, Fly Ash and Superplasticizer

**DOI:** 10.3390/ma15155369

**Published:** 2022-08-04

**Authors:** Chongchong Qi, Binhan Huang, Mengting Wu, Kun Wang, Shan Yang, Guichen Li

**Affiliations:** 1 China State Key Laboratory of Strata Intelligent Control and Green Mining Co-Founded by Shandong Province and the Ministry of Science and Technology, Shandong University of Science and Technology, Qingdao 266590, China; 2 School of Resources and Safety Engineering, Central South University, Changsha 410083, China; 3 School of Mines, China University of Mining and Technology, Xuzhou 221116, China

**Keywords:** concrete, blast furnace slag, fly ash, superplasticizer, principal component analysis, machine learning

## Abstract

Blast furnace slag (BFS) and fly ash (FA), as mining-associated solid wastes with good pozzolanic effects, can be combined with superplasticizer to prepare concrete with less cement utilization. Considering the important influence of strength on concrete design, random forest (RF) and particle swarm optimization (PSO) methods were combined to construct a prediction model and carry out hyper-parameter tuning in this study. Principal component analysis (PCA) was used to reduce the dimension of input features. The correlation coefficient (R), the explanatory variance score (EVS), the mean absolute error (MAE) and the mean square error (MSE) were used to evaluate the performance of the model. R = 0.954, EVS = 0.901, MAE = 3.746, and MSE = 27.535 of the optimal RF-PSO model on the testing set indicated the high generalization ability. After PCA dimensionality reduction, the R value decreased from 0.954 to 0.88, which was not necessary for the current dataset. Sensitivity analysis showed that cement was the most important feature, followed by water, superplasticizer, fine aggregate, BFS, coarse aggregate and FA, which was beneficial to the design of concrete schemes in practical projects. The method proposed in this study for estimation of the compressive strength of BFS-FA-superplasticizer concrete fills the research gap and has potential engineering application value.

## 1. Introduction

Concrete is a widely used building material containing a high proportion of artificially manufactured stone, generally mixed with cement, coarse aggregates (i.e., gravel or pebbles), fine aggregates (i.e., sand), admixture, and water. The strength of concrete is related to that of cement. However, cement has a high cost and is also the main cause of concrete’s environmental impacts. With the growth of the cement industry, carbon dioxide (${\mathrm{CO}}_{2}$) emissions are increasing, currently accounting for 5–10% of total global anthropogenic ${\mathrm{CO}}_{2}$ emissions; in addition, issues including high levels of energy consumption in the cement production process are increasingly emerging [1,2,3]. Although improving cement manufacturing technologies can help to reduce ${\mathrm{CO}}_{2}$ emissions [4], studies have increasingly shown that using alternative materials might be a more accessible and sustainable approach [2]. Therefore, with the proposal of the new concept of national green development of China and the increasing focus on simultaneously achieving both healthy living environments and development, green building materials are becoming more widespread in their use, especially solid waste produces such as blast furnace slag (BFS) and fly ash (FA), which are used as partial replacements to Portland cement [5].

BFS is a by-product of smelting pig iron at a temperature of approximately 1500 °C in a blast furnace. Smelting iron ore with a grade of 60–65% yields 0.3–0.5 tonnes of slag per tonne of pig iron in most cases [6]. According to previous research, China produced 32% of the world’s slag in 2017, totaling more than 139 million tonnes [7]. The huge production and buildup of BFS can pollute the environment, while the natural resources remaining in BFS may be wasted, potentially limiting the steel industry’s ability to expand. In China, the total usage rate of BFS is estimated to exceed 85%. It has two primary uses in concrete preparation: as a light concrete aggregate and as a cement substitute. The reaction of activated BFS is comparable to that of Portland cement due to the presence of SiO_2_, CaO and Al_2_O_3_ in its major composition [8]. Because of its pozzolanic reaction and hydraulic activity, BFS has been utilized as a primary supplemental cement ingredient for over a century [9,10]. Abdul Razak B. H. et al. [11] demonstrated that BFS represents a good alternative to Portland cement in terms of decreasing hydration heat and increasing durability while also maintaining the concrete’s strength. In engineering practice, due to its high cement content, the overall heat output is significant when a considerable volume of concrete is poured at once. However, this heat release may be significantly reduced by using large amounts of BFS instead of cement, thus lowering the risk of thermal cracking. Furthermore, high levels of BFS are typically used in concrete formulations for maritime applications because they reduce chlorine penetration, ensuring greater protection of the reinforcing steel [10].

FA is a fine solid particle type formed by pulverized coal entering the furnace at ~1300–1500 °C and cooling after absorbing heat from the hot surface under the suspended combustion condition. The major sources of FA are coal-fired power plants and urban central heating boilers, which account for 60–88% of the by-products of pulverized coal combustion. As the power industry has developed, FA emissions from coal-fired power plants have increased year on year, becoming among the main industrial waste residue emission types in China. In 2016 and 2017, the emissions of FA in China reached 655 million tons and 686 million tons, respectively; these emissions cause environmental pollution, with the heavy metals contained within being particularly damaging to both vegetation and humans. Based on the pozzolanic effect and the cementitious properties of FA, this material can be used as a binder or raw material for producing clinker and partial replacing cement in concrete production [12]. Compared to Portland cement, FA-based concrete has lower water demands, less hydration heat, less risk of early-age cracking, and high late strength gain [13].

At present, studies of BFS and/or FA-based concrete types are mainly focused on their mechanical properties, especially the elastic modulus, compressive strength, and tensile strength parameters. In 1998, M.N. Haque found that when the maximum content of FA is limited to less than 10%, the highest concrete performance can be achieved, with its strength greatly improving over time. Oner et al. [14] performed a laboratory-based study in which a total of 28 concrete mixtures with different formulations were prepared and maintained; their study concluded that up to 40% of cement can be replaced by FA without reducing the resulting concrete’s mechanical properties. S.E. Chidiac et al. [15] investigated the mechanical characteristics of concrete incorporating ground granulated BFS (GGBFS) and identified that the curing time needed to attain the same compressive strength of BFS cement (BFSC) as that of ordinary Portland cement (OPC) was more than double that of OPC. When the cementitious material composition is the same, the compressive strength after 28 days is comparable to regular PC concrete when GGBFS is utilized at up to 50% content [16]. The strength at 28 days may be smaller than the 28-day strength of OPC concrete when the GGBFS content is greater than 50% [10]. Based on the principle that the higher the specific surface area, the better the hydration process for cementitious materials, Subpaasa Prang et al. demonstrated that increasing the Blaine value of BFS boosts the concrete’s compressive strength [17]. As a result, while producing BFSC, more finely grinding is among the primary techniques to effectively improve the resulting concrete’s strength [18].

As previously noted, concrete strength is the primary requirement for concrete designed with SCMs, which can be determined through extensive laboratory experiments. However, lab-based investigations are not only costly but also time-consuming, hindering the efficient design of concrete with SCMs. Accordingly, a more efficient strength prediction method is desirable.

The development of artificial intelligence makes accurately predicting concrete strength viable [19]. For example, Nazanin et al. utilized five high-precision artificial neural networks, including radial basis function, multilayer perceptron, support vector regression, adaptive network-based fuzzy inference system (ANFIS), and deep neural network approaches, to predict the elastic modulus and compressive and tensile strengths of FA-based concrete. The estimated mechanical properties showed strong agreement with experimental results ($R^{2}$ > 0.98) [20]. Deepak Choudhary et al. employed an artificial neural network (ANN) in combination with sequential feature selection (SFS) to predict the compressive strength of fly ash-based concrete ($R^{2}$ = 0.991). The SFS approach was used to identify the relevant components with the greatest influence on the compressive strength, which were found to be mainly cement, silica fume, FA, and water [21]. M.I. Waris et al. combined image processing, ANN, and ANFIS methods to predict the mechanical properties of hybrid concrete [22]. Despite these successes, to date, predicting the strength of concrete made with BFS, FA, and superplasticizer has been rarely studied. In addition, the influence of dimensionality reduction on ML performance has also not been comprehensively investigated.

In this context, we applied ML techniques that combine random forest (RF) and PSO approaches for predicting the compressive strength of concrete made with BFS, FA, and superplasticizer. The proposed method uses RF for non-linear relational mapping from inputs (i.e., cement, BFS, FA, water, superplasticizer, coarse aggregate, fine aggregate, and age) to the output (concrete compressive strength). Principal component analysis (PCA) was also used for dimensionality reduction. The model was applied to a dataset collected for this experiment containing 1030 samples, including eight component variables of concrete: cement, BFS, FA, water, superplasticizer, coarse aggregate, fine aggregate and age. Prediction accuracy was assessed using performance measures and sensitivity analysis was also performed.

## 2. Materials and Method

### 2.1. ML Modelling Framework

**Figure 1:**
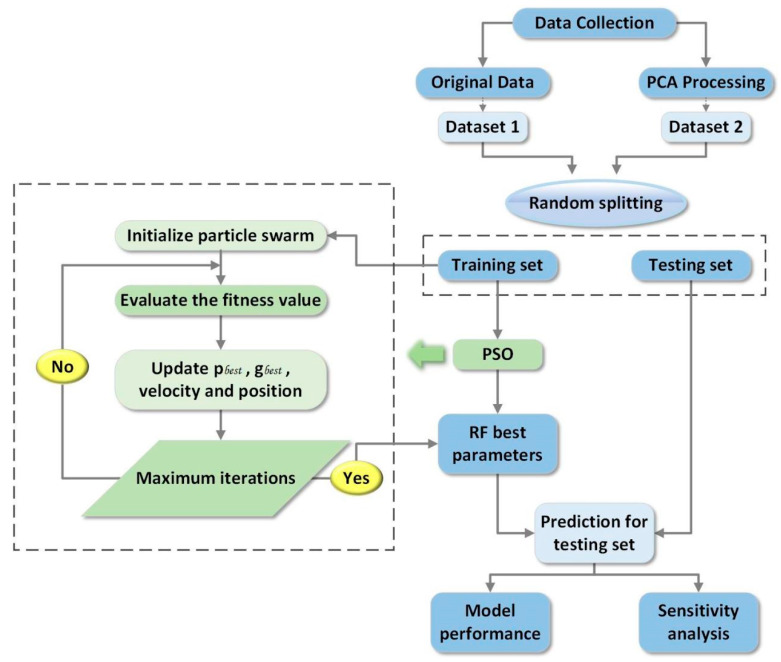
The overall procedure for concrete strength estimation using the RF_PSO.

To forecast the compressive strength of concrete, we employed the RF method in combination with PCA and hyper-parameter optimization, as shown in Figure 1.

The modeling framework can be summarized as follows. The initial step involved collecting data from previous studies, followed by dimension reduction using the PCA approach. The third stage involved randomly dividing the dataset and evaluating model performance to determine the optimal splitting ratio. The fourth stage involved applying the PSO approach to optimize the RF hyper-parameters to construct the optimized RF models containing the best parameters. In the fifth stage, evaluation metrics were employed to evaluate the model’s performance, followed by the sixth stage in which a feature sensitivity study was conducted.

### 2.2. Prediction Models

Artificial intelligence is a branch of computer science and technology based on multiple experimental experiences and obtaining knowledge and learning strategies. Its emergence greatly reduces the time and cost involved in engineering tasks, and improves the computational efficiency of engineering tasks, especially those involving high-dimensional problems. The variables in this study interact with one another, for example, cement, FA, and BFS all react with water and these reactions affect one another, but the effects of these interactions are extremely difficult to calculate. This is true in many technical challenges. As a result, artificial intelligence provides considerable benefits in the field of engineering.

In this study, the variables influencing the magnitude of the compressive strength of concrete are interdependent. When one of the variables changes, the other variables will also produce correspondingly different changes in response. Thus, to investigate the effect of the different components on the strength of concrete in this study, regression analysis was conducted to establish the regression variance and predict how values follow their corresponding variables. Due to the non-linear relationship between concrete strength and the studied variables, the required calculations would be extremely time-consuming and complex if performed manually; however, artificial intelligence represents an ideal approach for multivariable concrete strength modeling. In this study, the RF algorithm was selected for non-linear relationship modeling, PSO was used to optimize the RF hyper-parameters, and PCA was used to evaluate the influence of dimensionality reduction on modeling performance.

#### 2.2.1. Random Forest

RF is a reliable and powerful machine learning algorithm, proposed by Leo Breiman and Adele Cutler [23]. The RF is a classification and regression algorithm that belongs to the bagging (i.e., bootstrap aggregation) algorithm in integrated learning [24]. RFs are characterized by decision trees (DTs), in which a model is constructed based on a randomized training set; the values of the different DTs are not correlated and are calculated independently, and the average of the results obtained using these decision trees is used in the prediction process [25,26,27,28,29].

**Figure 2:**
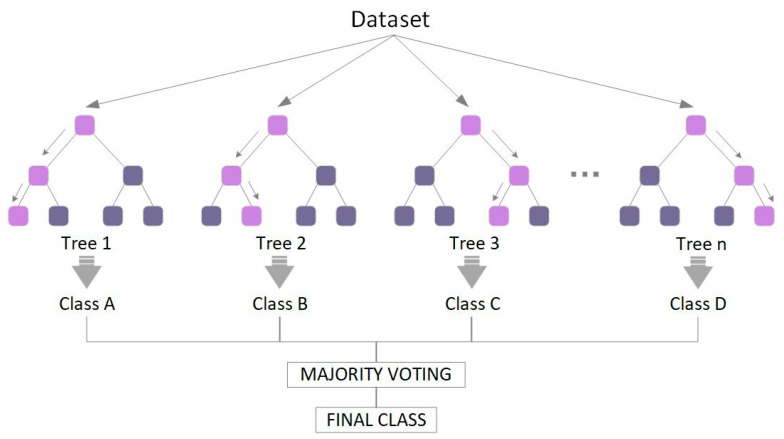
A typical architecture of RF. Note that the light purple block represents the best feature of the selected segmentation node.

A random selection of samples is released from the training data during the construction of a DT, as shown in Figure 2—rather than using all the data’s characteristics, some are chosen at random for training. Each tree utilizes various samples and features, and the training outcomes are organically varied. In this random feature selection approach, no prior information is provided about which samples are anomalous or which characteristics have a strong influence on classification results [30]. Thus, the random feature selection approach decreases the impact of both these aspects on modeling outcomes. Because the accuracy of RF is generally higher than that of DTs alone when solving complex problems, it is frequently applied in classification and regression contexts.

The advantages of RF are as follows, which contributed to its selection as the approach used in the current study:

1. High accuracy can be achieved by using an integrated algorithm.
2. The random process (i.e., random sampling and random features) reduces the over-fitting of a single DT, enables the processing of high-dimensional data with more features, and does not involve feature selection.
3. The inclusion of unusual data has minimal impact on the outcomes.
4. Multiple DTs are independent of one another and their computation times are short [31].

#### 2.2.2. Principal Component Analysis

PCA is a statistical algorithm that use in data analysis [32,33]. The dimensionality of the data is reduced by preserving the feature dimension with the largest variation and rejecting the feature dimension with nearly no variation. The PCA method was first introduced by Pearson for non-random variables and was then extended to random vectors and the data in which is translated from the old coordinate system to the new one using a simple linear algebraic derivation.

In many research fields and applications, it is commonly necessary to analyze large amounts of data and identify patterns through statistics. However, due to the correlation between variables, that is, there is some information overlap between the responses of different variables, which increases the complexity of the research. Therefore, principal component analysis aims to reduce the complexity of the dataset by removing duplicate variables (i.e., highly correlated variables) from the original variables and creating as few new orthogonal variables as possible [34] (i.e., principal components [32]), while retaining as much of the original information as possible. There is no correlation between these new orthogonal variables [35,36]. PCA is a widely used, simple, and adaptable tool for descriptive data analysis; these attributes make it useful for application to a wide range of situations and data types across many disciplines and, thus among the best methods for dimensionality reduction [37].

#### 2.2.3. Particle Swarm Optimization (PSO)

**Figure 3:**
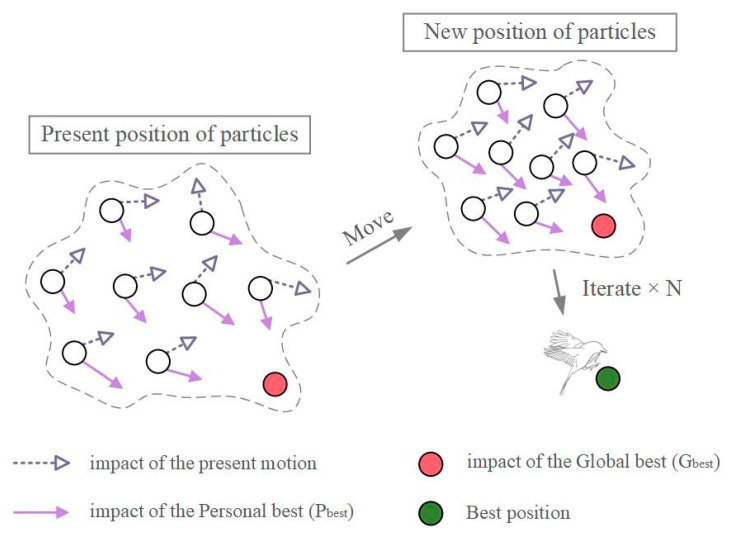
A typical architecture of PSO.

In essence, PSO comprises two main components: artificial life and evolutionary computation. PSO creates massless particles based on the predatory behavior of a flock of birds. Each particle changes its speed and movement direction by scanning the search space and sharing its current individual value with other particles, as shown in Figure 3. The particle maintains its location in each iteration by using the individual poles it has discovered, with the global poles identified by the transmission of information between particles. The optimum location is eventually identified after many iterations [38].

As an evolutionary computing technique, PSO adjustment toward $G_{\mathrm{best}}$ is similar to the crossover operation utilized by genetic algorithms (GA) but is far more straightforward than GA. PSO overcomes GA’s high c complexity and is far less computationally intensive than GA to achieve the same high-quality solution [39].

The implementation of PSO in artificial intelligence is both simple and comprehensive and is highly useful for both scientific research and engineering applications [40]. Previous studies have demonstrated that PSO can improve model accuracy, thus this approach is currently commonly employed for optimization problems in concrete prediction models [41].

### 2.3. Dataset Preparation

#### 2.3.1. Dataset Sources

**Figure 4:**
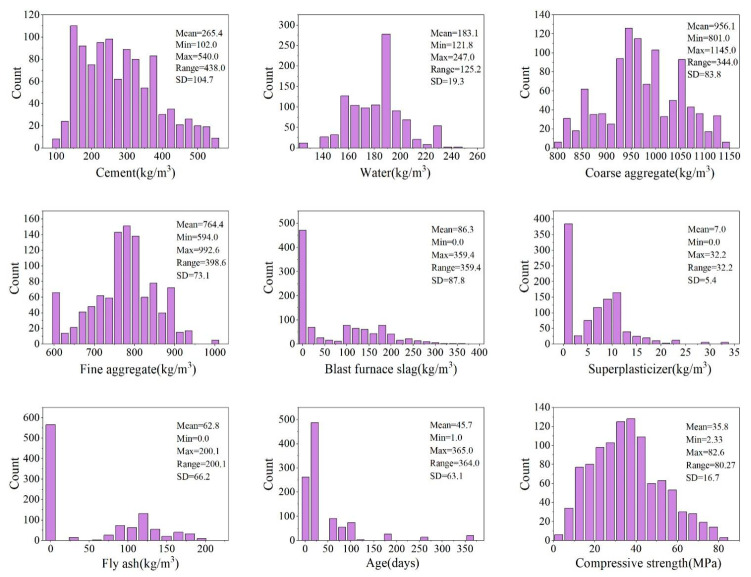
Histogram statistics for input and output variables.

When establishing a predictive model, the general premise involves collecting a representative dataset to ensure the accuracy and generalization ability of the model. In this study, a dataset of 1030 concrete compressive strength samples were collected. The samples were cured at a temperature of 20 ± 2 °C and humidity of not less than 95%, and the maximum aggregate size was 20 mm. The input variables for concrete strength include cement ($\mathrm{kg}/m^{3}$), blast furnace slag ($\mathrm{kg}/m^{3}$), fly ash ($\mathrm{kg}/m^{3}$), water ($\mathrm{kg}/m^{3}$), superplasticizer ($\mathrm{kg}/m^{3}$), coarse aggregate ($\mathrm{kg}/m^{3}$), fine aggregate ($\mathrm{kg}/m^{3}$), and age (days). The actual compressive strength for concrete of a given age is obtained by performing a typical laboratory-based compressive test procedure on a bulk specimen, with the data presented in raw form, i.e., not to scale. The performance of the materials used to prepare concrete samples in this study is in line with the standard specifications. The statistical analysis of the mean value, minimum value, maximum value, range and standard difference of each parameter is shown in Table 1 and presented in the form of histogram in Figure 4. This dataset is considered representative of concrete behavior and has been used in other ML algorithm studies [42].

#### 2.3.2. Dataset Pre-Processing

To evaluate the influence of dimensionality reduction on modeling performance, the original eight input variables were processed using PCA to transform them into a new linear combination. To ensure the reliability of the reduced dimensional information and the accuracy of the model predictions, the original features were projected as far as possible toward the dimension with the maximum amount of projected information, with at least 95% of the information preserved and the final five input variables being retained. Due to the orthogonalization of the input dataset, the correlation between the dependent and independent variables is better than that of the original data. However, the PCA-derived variables have no direct physical meaning—this approach is intended for data processing only and the interpretation of its meaning is therefore often somewhat more ambiguous and less complete than the original sample.

At this stage of the analysis, it is unclear whether the effect of dimensionality reduction is positive or negative on modeling performance. Accordingly, two datasets, denoted as dataset 1 (original dataset; eight inputs) and dataset 2 (after PCA processing; five inputs) were prepared and the model performance on both was compared.

#### 2.3.3. Dataset Division

In ML algorithms, the original dataset is commonly split into two parts. Data from the training set are used to train the model by determining the mapping from inputs to the output. The testing set is used to evaluate the model’s performance by testing the accuracy of the predictions of the already trained model on unknown data. In this study, we split the entire dataset into a training set and a testing set by random sampling according to a dataset division ratio.

Differences in the size and proportion of the training and test sets affect the performance and accuracy of the model [43]. To achieve the optimal dataset splitting ratio, the testing set size was progressively increased from 10% to 65% of the total data. The variations in modeling performance with changing test set size are expressed by R, with values closer to 1 indicating better correlation. The training–testing evaluation was repeated 50 times for each testing set size to reduce the potential influence of dataset sampling on the comparison.

**Figure 5:**
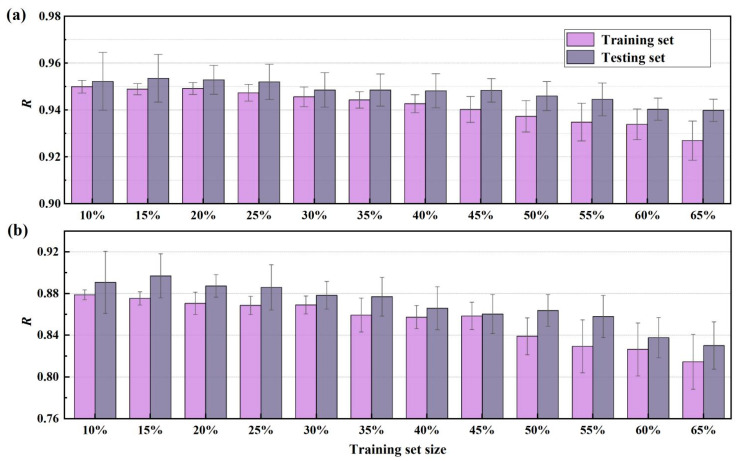
Influence of testing set size on the RF performance: (**a**) dataset 1 and (**b**) dataset 2.

As shown in Figure 5, the average R for the testing set first increases as the test set size increases from 10% to 15% and then decreases slightly with further testing set size increases up to 65%. The average R achieved on the training set decreases continuously as the test set size increases. To maintain consistency across all datasets and to optimize test performance, the testing set size was set at 15%.

### 2.4. Hyper-Parameter Training

Model parameters and hyper-parameters are the two parameter types used in ML models. Data estimation or data learning can be used to adjust the model’s parameters [44]. Hyper-parameters, unlike model parameters, are established before the model begins to learn and thus cannot be modified via training [45]. As a result, the selection of hyper-parameters significantly influences the model’s performance [46].

**Figure 6:**
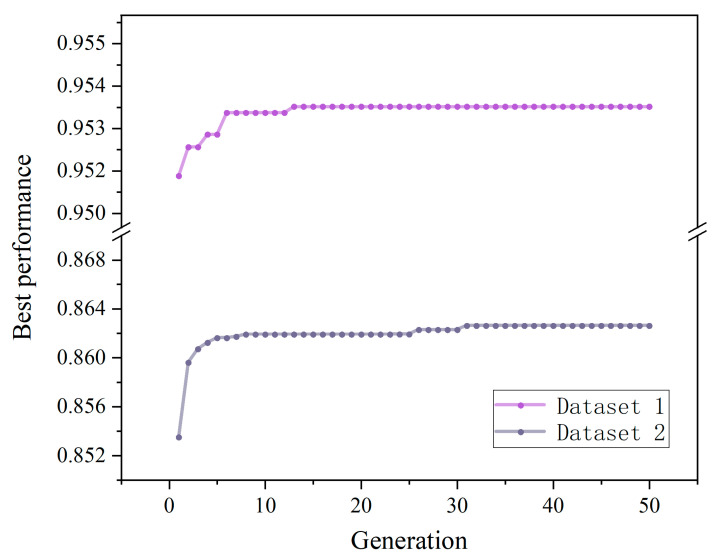
Evolution of $G_{\mathrm{best}}$ with PSO generations on two datasets.

In this paper, PSO is utilized to tune the hyper-parameters of RF for both datasets. To obtain the final hyper-parameter values, we optimized the RF hyper-parameters in a wide range (Table 2). Figure 6 demonstrates the evolution of $G_{\mathrm{best}}$ (global best) versus PSO generation, where the correlation coefficient R was used as fitness to compare with the optimal location $G_{\mathrm{best}}$ and the maximum number of iterations was set to 50.

It can be observed that with the increase in iteration times, fitness value kept increasing and the global optimal location $G_{\mathrm{best}}$ was constantly updated. After the R value increased from 0.9515 in the first generation to 0.9534 in the 15th generation, the R value tended to fluctuate steadily with the increase in iterations. As a result, PSO got the global optimal solution and the model had the best performance. The ideal hyper-parameters determined are shown in Table 2.

### 2.5. Performance Measures

In ML modeling, the model performance is evaluated by performance indicators. In this paper, four performance measures are used to validate the performance of the trained ML models: R, the explained variance score (EVS), the mean absolute error (MAE), and the mean squared error (MSE).

1. The correlation coefficient I [47]: also known as the Pearson correlation coefficient, was formulated by the statistician Karl Pearson [48]. In this study, the degree of linear correlation between the actual and estimated compressive strength values is represented by R. The R-value has an absolute value range of 0–1. [49]; the closer it is to 1, the more accurate the model is at forecasting. This parameter is defined as follows:

$$R=\frac{\sum^{\;}\left(x_{i}-\;\hat{x}\right)\left(y_{i}-\;\hat{y}\right)}{\sqrt{\sum^{\;}{\left(x_{i}-\;\hat{x}\right)}^{2}\sum^{\;}{\left(y_{i}-\;\hat{y}\right)}^{2}}}$$

where $x_{i}$ denotes the observed CS value, $\;\hat{x}$ is the mean of the observed value, $y_{i}$ is the predicted CS value of RF model, and $\;\hat{y}$ is the mean of the predicted value.

2. The EVS is the variance score used to explain the regression model. This metric measures the dispersion of errors in a dataset by comparing the variance of the errors in the dataset with the variance of the actual values in the dataset. The range of EVS values is [0,1], with values closer to 1 indicating more similar dispersion between the predicted and actual values; this scenario indicates that the results obtained through the model will be better at explaining the variance of the input variables, whereas smaller values indicate poorer results.

$$\mathrm{EVS}=1-\frac{\mathrm{Var}\left\{x_{i},y_{i}\right\}}{\mathrm{Var}\left\{x_{i}\right\}}$$

3. MAE [50]: The MAE parameter is calculated by averaging the sum of the absolute difference values between the actual and predicted values of compressive strength at all data points, thereby assessing how close the predicted results are to the true dataset. A smaller MAE value thus indicates a better model fit. This parameter is expressed as:

$$\mathrm{MAE}=\frac{1}{n}\sum_{i=1}^{n}\left|x_{i}-y_{i}\right|$$

4. MSE: The MSE parameter is the most commonly used evaluation metric in regression models. This metric calculates the mean of the sum of squares of the errors between the fitted data and the original data corresponding to the sample points. The closer the MSE is to zero, the more accurate the model.

$$\mathrm{MSE}=\frac{\sum_{i=1}^{n}\left(x_{i}-y_{i}\right)}{n}$$

## 3. Results and Discussion

In this study, the RF-PSO model was used to predict concrete strength by combining RF with PCA and PSO techniques. To reduce the dimensionality of the input variables before applying the RF, the PCA approach was employed for dimension reduction. The data were split randomly into two subsets: a training set and a testing set, comprising 85% and 15% of the total data, respectively, based on a sensitivity analysis. PSO was used to optimize the model’s hyper-parameters to achieve the optimum model performance.

### 3.1. Performances of Machine Learning Models

**Figure 7:**
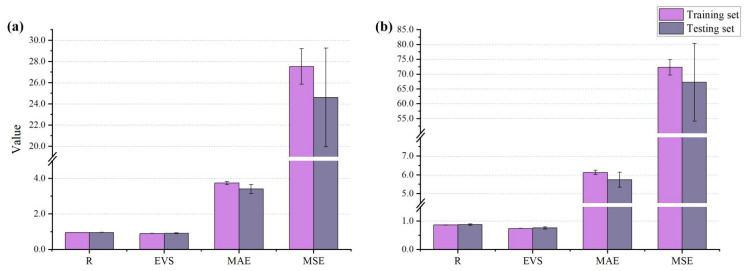
Performance measures for: (**a**) dataset 1 and (**b**) dataset 2.

Figure 7 shows the prediction accuracy of the RF model on datasets 1 and 2. The R, EVS, MAE, and MSE values between the observed and predicted values of the RF model were 0.954, 0.901, 3.746, and 27.535, respectively, on the original dataset (Figure 7a). These outcomes suggest that the RF model had excellent predictive capability on the original dataset. In contrast, the predictive performance on the PCA-processed dataset was not as good as on the original dataset, with values of R = 0.864, EVS = 0.740, MAE = 6.130, and MSE = 72.351. The above results indicate that dimensionality reduction using PCA negatively affected the modeling performance. In other words, dimension reduction appears to be unsuitable for the concrete dataset used in the present study.

**Figure 8:**
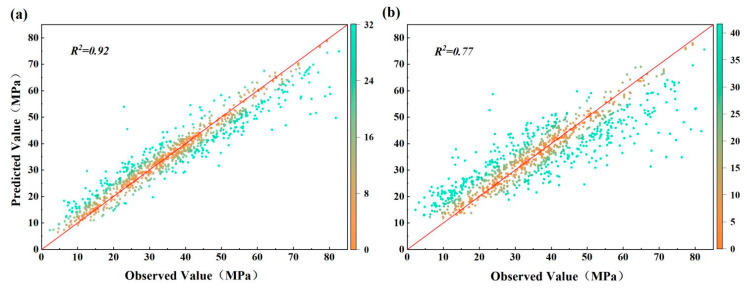
Comparison between observed and predicted strength values. (**a**) of dataset 1 and (**b**) of dataset 2.

Figure 8 shows a comparison between the RF model’s predicted and observed concrete compressive strength values reflected by the difference between the observed value and the predicted value. The closer the difference is to 0 (i.e., the closer the data distribution to the diagonal line), the better the prediction. For dataset 1, a total of 20.68% of the error data were lower than 1 MPa, 31.94% were in the range of 1–3 MPa, 23.40% were in the range of 3–5 MPa, and 23.98% were over 5 MPa, yielding an R2 value of 0.92 for the RF model (Figure 8a). In terms of dataset 2, a total of 12.91% of the error data were lower than 1 MPa, 24.56% of the error data were in the range of 1–3 MPa, 16.99% of the error data were in the range of 3–5 MPa, and 45.54% of the error data were higher than 5 MPa, producing an R2 value of 0.78 for the RF model (Figure 8b). The above results indicate good predictive performance of the RF models, with a better predictive performance recorded on the original dataset.

**Figure 9:**
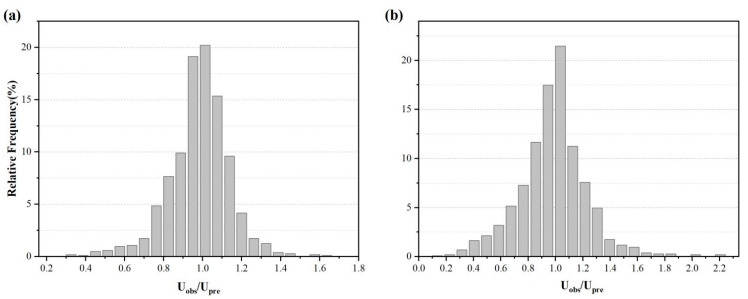
Relative frequencies of observed and predicted compressive strength ratios ${\mathrm{CS}}_{\mathrm{obe}}$/${\mathrm{CS}}_{\mathrm{pre}}$: (**a**) of dataset 1 and (**b**) of dataset 2.

Figure 9 shows histograms of the observed/predicted compressive strength (${\mathrm{CS}}_{\mathrm{obe}}$/${\mathrm{CS}}_{\mathrm{pre}}$) values using the optimal RF model on datasets 1 and 2. The frequency of ${\mathrm{CS}}_{\mathrm{obe}}$/${\mathrm{CS}}_{\mathrm{pre}}$ in the range of 0.8–1.2 on both datasets were above 80%, indicating that the optimal RF model performed well. In addition, both datasets’ histograms plot slightly to the right of 1, indicating that the optimum RF models predict slightly lower strength values than the true experimental values.

### 3.2. Sensitivity Analysis of Input Variables

**Figure 10:**
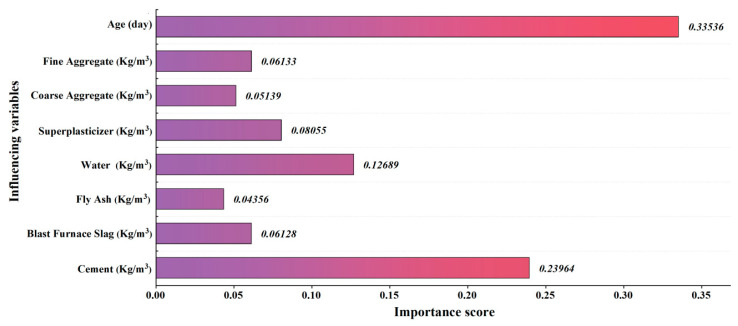
Sensitivity analysis of input variables.

Having obtained an accurate compressive strength prediction model, it is crucial to understand which variables have a major impact on the compressive strength of high-performance concrete. Sensitivity analysis was performed in this study by changing one input variable at a time while keeping the others constant [51]. The predicted output was recorded as the specific variable was changed—the greater the effect of the changing variable on the output, the higher the importance score of that variable. The same procedure was repeated for each of the input variables. After obtaining the importance scores for all inputs, the values were normalized so that their summation was equal to 1, with the normalized importance scores shown in Figure 10. The authors note here that a sensitivity study was not performed on dataset 2 as the PCA-processed variables do not have any physical meaning.

As shown, the most important variable was identified as curing age, which contributed 33.536%, followed by cement (23.964%) and water (12.689%). Superplasticizer, fine aggregate, BFS, coarse aggregate, and FA contributed relatively less, with values of 8.055%, 6.133%, 6.128%, 5.139%, and 4.356%, respectively. On this basis, the influence of BFS, FA, and superplasticizer on concrete strength was explained as follows.

BFS improves the mechanical characteristics of concrete by reducing its porosity and increasing its resilience to weak acids and salts [52]. In comparison to OPC concrete, BFS concrete takes longer to hydrate. The compressive strength of concrete is highest when BFS substitutes 10% of the cement, according to Kishan Lal Jain et al. [53].

The pozzolanic effect and cementitious properties of FA are primarily responsible for the influence of FA on concrete strength. Young Keun Cho et al. reported that after 91 days of curing time, the strength of FA-based concrete increased due to the pozzolanic effect [54]. In short, the replacement of cement by FA will rend to result in a decrease in early strength but a gain in long-term strength [55]. For FA dosage, the maximum percentage of DA used to replace OPCs is ~35–45% [56,57]. A previous study found that by combining FA with BFS, it is feasible to counteract FA-based concrete’s short-term strength loss while maintaining long-term performance [58].

Several studies have shown that adding superplasticizer to concrete decreases the quantity of water required for mixing, thus lowering the water–cement ratio and porosity and improving the superplasticizer concrete performance [59,60,61]. Superplasticizers can also increase the flowability of the produced concrete slurry and help to prevent the concrete’s characteristics from deteriorating due to extended mixing durations [62]. The amount of superplasticizer is usually kept to less than 3%, which will produce a marginal improvement in the concrete’s compressive strength [63].

## 4. Conclusions and Outlook

In this study, ML techniques were used to construct a model for the precise and rapid prediction of the concrete strength. The influences of BFS, FA, and superplasticizer on concrete strength were key target variables during dataset collection. The content of cement, water, coarse aggregate, and fine aggregate, as well as age and a variety of chemical additives, were selected as model inputs. The RF algorithm was utilized in combination with PCA and PSO for the concrete strength prediction. The specific conclusions are as follows:

(1) The R, EVS, MAE and MSE values on the original dataset were 0.954, 0.901, 3.746 and 27.535, respectively, indicating that the ML model constructed in this study can accurately predict the strength of concrete prepared with BFS, FA, and superplasticizer, which has potential engineering application value.
(2) After PCA processing, the prediction accuracy decreased (R = 0.864, EVS = 0.740, MAE = 6.130, MSE = 72.351), indicating that PCA dimension reduction has a negative impact on ML modeling and cannot be adopted. However, there is no doubt that the combination of the two has exploratory significance.
(3) The sensitivity analysis showed that curing time has the greatest influence on the compressive strength of concrete, followed by cement > water > superplasticizer> fine aggregate > blast furnace slag > coarse aggregate > fly ash. This provided potential ideas for further improving the strength of concrete.

However, this study still had some limitations, such as the size of the dataset on the compressive strength of concrete was still a little small to be representative. The positive and negative effects of cement, water and superplasticizer on compressive strength have not been specified, and how to improve the compressive strength of concrete based on post-model analysis also needed to be further explored.

## Figures and Tables

**Table 1:** The statistical analysis in the compressive strength tests.

Parameter	Unit	Type	Mean	Minimum	Maximum	Range	SD
Cement	$\mathrm{kg}/m^{3}$	Input	265.4	102.0	540.0	438.0	104.7
Water	$\mathrm{kg}/m^{3}$	Input	183.1	121.8	247.0	125.2	19.3
Coarse aggregate	$\mathrm{kg}/m^{3}$	Input	956.1	801.0	1145.0	344.0	83.8
Fine aggregate	$\mathrm{kg}/m^{3}$	Input	764.4	594.0	992.6	398.6	73.1
Blast furnace slag	$\mathrm{kg}/m^{3}$	Input	86.3	0.0	359.4	359.4	87.8
Superplasticizer	$\mathrm{kg}/m^{3}$	Input	7.0	0.0	32.2	32.2	5.4
Fly ash	$\mathrm{kg}/m^{3}$	Input	62.8	0.0	200.1	200.1	66.2
Age	days	Input	45.7	1.0	365.0	364.0	63.1
Compressive strength	MPa	Output	35.8	2.33	82.6	80.27	16.7

**Table 2:** Tuned RF hyper-parameters and their tuning outcome.

Hyper-Parameters	Explanation	Type	Tuning Range	Dataset 1	Dataset 2
Max_depth	The maximum depth of each DT	Integer	1–15	15	15
Number_DT	The number of DTs in the forest	Integer	50–2000	1457	356
Min_samples_split	The minimum number of samples required to split an internal node	Integer	2–15	2	2
Min_samples_leaf	The minimum number of samples at the leaf node	Integer	1–15	1	1
Max_features	The number of features to be used when looking for the best split.	Float	0.4–1	0.466	0.978

## Data Availability

The data will be available upon request.
